# Autonomous motivation moderates the relationship between fitness application usage and exercise behavior through flow experience: a cross-sectional study among Chinese university students

**DOI:** 10.3389/fspor.2026.1877847

**Published:** 2026-06-22

**Authors:** Jingjing Song, Jiaying Li, Dan Li

**Affiliations:** 1 College of Physical Education and Health Management, Chongqing University of Education, Chongqing, China; 2 School of Physical Education, Chongqing University of Posts and Telecommunications, Chongqing, China; 3 School of Physical Education, Wuhan University of Science and Technology, Wuhan, Hubei, China

**Keywords:** autonomous motivation, exercise behavior, fitness applications, flow experience, moderated mediation, university students

## Abstract

**Background:**

While mobile fitness applications have proliferated globally, their effectiveness in promoting physical activity among university students remains inconsistent, with high attrition rates reported. Understanding the psychological mechanisms underlying application usage and exercise behavior is crucial for optimizing digital health interventions. This study examined a moderated mediation model investigating how autonomous motivation regulates the relationship between fitness application usage and exercise behavior through flow experience among Chinese university students.

**Methods:**

A cross-sectional survey was conducted with 350 university students (56.0% male, mean age 20.4 years) actively using fitness mobile applications in China. Participants completed validated questionnaires measuring fitness application usage, flow experience, autonomous motivation, and physical activity levels (assessed via International Physical Activity Questionnaire). Data were analyzed using hierarchical regression analysis and bootstrapping procedures with 5,000 resamples to test the moderated mediation model.

**Results:**

The moderated mediation model demonstrated excellent fit, explaining 74.3% of variance in flow experience and 46.9% in exercise behavior. Autonomous motivation significantly moderated the relationship between application usage and flow experience (β=0.423, p<.001), with stronger effects observed at higher motivation levels. Flow experience significantly mediated the relationship between application usage and exercise behavior [indirect effect = 0.318, 95% confidence interval (0.254, 0.384)], accounting for 60.4% of the total effect. The index of moderated mediation was significant [0.225, 95% confidence interval (0.162, 0.292)], indicating that the indirect effect was 5.84 times stronger in high vs. low autonomous motivation groups. Simple slope analyses revealed that at high motivation levels, application usage strongly predicted flow experience (β=1.021, p<.001), whereas this effect was attenuated at low motivation levels (β=0.175, p<.001).

**Conclusions:**

Fitness applications were associated with exercise behavior primarily through flow experiences, with this association being significantly amplified among students with higher autonomous motivation. These findings suggest that application developers should prioritize features fostering intrinsic motivation and optimal experiences, while interventions should target motivation enhancement to maximize application effectiveness in supporting sustained physical activity among university populations.

## 1 Introduction

Globally, the continued decline in physical activity among university students has become a prominent public health concern. A pooled analysis of 358 population-based surveys encompassing 1.9 million participants reported little improvement in the prevalence of insufficient physical activity among adults worldwide between 2001 and 2016, with fewer than 40% meeting recommended activity levels [1]. The situation appears particularly challenging in university populations. This developmental period represents a critical window for the consolidation of lifestyle habits: structured physical education requirements typical of secondary school have ended, and responsibility for sustaining an active lifestyle shifts largely to self-management [2, 3]. Behavioral patterns established during this transition often persist into later adulthood, shaping long-term trajectories of either active or sedentary living.

Regular physical activity is closely linked to cardiovascular health, metabolic functioning, emotion regulation, and broader psychological well-being [4, 5]. Evidence syntheses further indicate that maintaining an active lifestyle is associated with a 20%–30% reduction in the risk of chronic disease [6]. Nevertheless, adherence to exercise remains a persistent challenge for many young adults. A longitudinal study of fitness center members found that more than half discontinued their exercise program within six months of initiation [7]. Such high dropout rates raise a central question for both research and practice: which factors facilitate the sustained maintenance of exercise behavior?

The rapid expansion of mobile fitness applications (apps) offers a promising entry point to address this challenge. The widespread adoption of smartphones has reshaped how individuals access health information, track behavior, and engage with interventions [8, 9]. Contemporary fitness apps can deliver personalized training guidance, real-time performance feedback, and socially mediated support directly to users [10]. A systematic review and meta-analysis of nine randomized controlled trials reported that app-based interventions increased daily step counts by approximately 477 steps, with stronger effects observed in short-term programs (≤3 months) [11]. These findings suggest that apps may be associated with initiating exercise behavior. However, their long-term impact remains uncertain. A secondary analysis of an mHealth intervention reported that nonusage attrition is widespread among adolescents, with a substantial proportion discontinuing app use within weeks of download [12]. This common “early uptake–rapid decay” pattern points to a deeper theoretical issue: technology may be a necessary enabling condition, but it is unlikely to be sufficient for durable behavior change. Accordingly, identifying the psychological mechanisms that determine sustained effectiveness—and explaining why individuals respond heterogeneously to the same digital tool—has become a key agenda for research in digital health and exercise psychology.

### 1.1 Fitness app use and flow experience

The functional architecture of mobile fitness applications aligns closely with the conditions for optimal experience described in flow theory. Flow refers to a qualitative shift in consciousness in which individuals become fully absorbed in an activity, experiencing a merging of action and awareness, altered time perception, and intrinsic reward derived from the activity itself [13, 14]. Nakamura and Csikszentmihalyi identified three core antecedents of flow: clear goals, immediate feedback, and a dynamic balance between challenge and skill [14]. Fitness apps are well positioned to operationalize these conditions. They typically provide explicit goal setting, real-time performance feedback, and adjustable training difficulty. When users complete an exercise set, instant displays of heart rate changes, caloric expenditure, and completion percentages strengthen the perceived link between action and outcome. At the same time, adaptive difficulty can help keep task demands within an optimal range—neither so high as to elicit anxiety nor so low as to induce boredom—thereby supporting the emergence of flow.

In *Flow in Sports*, Jackson and Csikszentmihalyi [15] described how flow manifests in physical activity, emphasizing a sense of precise control over bodily movements, intense concentration on the ongoing process, and deep enjoyment inherent in exercise. A defining feature of this experience is its autotelic quality: exercise is pursued not primarily as an instrument for external outcomes (e.g., weight loss or health goals), but because it is experienced as inherently worthwhile. Evidence from elite sport further indicates a robust positive association between flow, performance, and sustained participation [16]. This qualitative shift is central to understanding exercise behavior: once exercise is experienced as something one wants to do rather than something one should do, persistence becomes less dependent on continual exertion of willpower or the provision of external rewards.

Applied to the fitness app context, this logic suggests that apps may facilitate exercise behavior by optimizing the goal–feedback–challenge structure and thereby creating situational conditions conducive to flow. Flow, in turn, functions as a positive subjective state that becomes a psychological resource for repeated engagement. In this sense, apps are not merely informational tools; they can also serve as media for cultivating intrinsically rewarding experiences, which may support sustained exercise participation. Accordingly, we propose:

**H1**: Flow experience mediates the association between fitness app use and exercise behavior. Specifically, greater fitness app use is associated with enhanced users’ flow experience during exercise, which in turn predicts exercise behavior.

### 1.2 The moderating role of autonomous motivation

Although fitness apps may provide external conditions supportive of flow, users are unlikely to benefit uniformly. Self-determination theory offers a useful framework for explaining such heterogeneity [17]. Ryan and Deci distinguish motivation by its quality, arguing that autonomous motivation—engaging in an activity because it is congruent with personal values, satisfies psychological needs, or is genuinely enjoyable—consistently outperforms controlled motivation in predicting behavioral initiation, intensity, and long-term maintenance [17]. A systematic review in the exercise domain further identifies autonomous motivation as one of the most reliable predictors of sustained participation in physical activity [18].

Motivational quality is also likely to shape how individuals interact with fitness apps. Students who use apps because they value and enjoy exercise may be more inclined to explore app functions deeply, experiment with training modes, and approach challenges with curiosity rather than apprehension—features that are conducive to flow. By contrast, individuals driven primarily by external pressures (e.g., appearance concerns or others’ expectations) or internal compulsion (e.g., guilt about not exercising) may treat the app as a monitoring device, completing tasks in a mechanical manner with limited engagement, thereby reducing the likelihood of entering a flow state. Put differently, apps may provide the opportunity for flow, but whether that opportunity is translated into an actual flow experience may depend on the user’s motivational orientation.

This proposition is consistent with Bandura’s account of person–environment interactions in social cognitive theory, which emphasizes that environmental influences are not determinative but operate through individuals’ cognitive appraisals and self-regulatory processes [19]. In a fitness app setting, app features (environmental factors) and users’ motivational characteristics (personal factors) jointly shape experiential quality and behavioral outcomes. Highly autonomously motivated users may not only attend to performance feedback, but also integrate it into their self-concept, deriving competence and meaning. Those with low autonomous motivation may perceive the same feedback as merely numerical information, eliciting limited affective resonance.

On this basis, we propose:

**H2**: Autonomous motivation moderates the relationship between fitness app use and flow experience. Specifically, the positive association between fitness app use and flow experience is stronger among students with higher autonomous motivation and weaker among those with lower autonomous motivation.

Integrating H1 and H2 yields a moderated mediation model: the indirect pathway from fitness app use to exercise behavior via flow experience varies as a function of autonomous motivation. Hayes’ framework provides a formal statistical rationale for such models, specifying that moderated mediation is supported when the magnitude of an indirect effect differs across levels of a moderator [20]. Therefore, we propose:

**H3**: Autonomous motivation moderates the indirect effect of fitness app use on exercise behavior via flow experience. This indirect effect is expected to be stronger at higher levels of autonomous motivation.

### 1.3 Study aims and significance

Despite the theoretical coherence of the proposed framework, empirical tests of this integrated model remain limited. Existing studies have often examined pairwise associations in isolation, such as links between app use and exercise behavior [21, 22], flow and exercise behavior [23], or motivation and exercise participation [24–26], with comparatively few efforts to evaluate all components within a unified analytic structure. Moreover, much of the available evidence is based on Western samples [24, 25]. The mechanisms through which fitness apps influence exercise behavior among Chinese university students—a large population facing distinctive academic demands—remain insufficiently documented.

The present study therefore aims to develop and test a moderated mediation model to clarify the interrelations among fitness app use, flow experience, autonomous motivation, and exercise behavior in Chinese university students. Beyond addressing whether app use is associated with exercise behavior, the model is designed to illuminate for whom the association is stronger (the moderating role of autonomous motivation) and through what process it operates (the mediating role of flow experience). Theoretically, the study advances understanding of the technology–experience–behavior linkage in digital health psychology and highlights motivational quality as a key moderator in person–technology interaction. Practically, the findings may inform the design and optimization of fitness apps for university students, particularly with respect to personalized feature configuration, user segmentation, and strategies to promote long-term engagement.

## 2 Methods

### 2.1 Participants

A convenience sampling strategy was used to recruit undergraduate and postgraduate students from a comprehensive university in China who were currently using mobile fitness applications (e.g., Keep, Yuepaoquan, Gudong, Mi Fitness). Eligibility criteria were: (1) being a current university student aged ≥18 years; (2) current use of at least one fitness app; and (3) providing electronic informed consent. Exclusion criteria were: (1) survey completion time <3 min or >60 min; (2) obvious patterned responding (e.g., selecting the same option throughout); (3) failure on an embedded attention-check item; and (4) self-report of not currently using a fitness app. A total of 350 questionnaires were returned. After data screening, all 350 cases were retained as valid responses (valid response rate: 100%). The sample comprised 196 men (56.0%) and 154 women (44.0%). Year of study was distributed as follows: first year (n=103, 29.4%), second year (n=85, 24.3%), third year (n=75, 21.4%), fourth year (n=68, 19.4%), and postgraduate or above (n=19, 5.4%). Participants represented a range of academic disciplines: science and engineering (n=117, 33.4%), economics/management (n=91, 26.0%), humanities and social sciences (n=59, 16.9%), medicine (n=38, 10.9%), arts/sports (n=36, 10.3%), and other fields (n=9, 2.6%). All participants reported current fitness app use. App use duration was: >1 year (n=96, 27.4%), 6–12 months (n=84, 24.0%), 3–6 months (n=68, 19.4%), 1–3 months (n=67, 19.1%), and <1 month (n=35, 10.0%). The mean duration score was 3.40 ± 1.33 on a 1–5 scale, with higher scores indicating longer use. Sample size adequacy was evaluated for mediation model testing. Assuming 80% power, a two-sided significance level of 0.05, and a medium effect size ($f^{2}=0.15$), G*Power 3.1 indicated a minimum required sample of 129; the achieved sample (N=350) exceeded this threshold, providing sufficient statistical power.

### 2.2 Procedure

Ethical approval was obtained from the Ethics Committee of Chongqing University of Education (Approval No.: CQUE-LL20260306001; approval date: March 6, 2026) prior to the commencement of data collection. The data were collected online via Wenjuanxing (a secure Chinese online survey platform) from March 7 to March 15, 2026. Before accessing the questionnaire, participants read an electronic informed consent form describing the study aims, procedures, voluntariness, confidentiality safeguards, potential risks and benefits, and researcher contact information. Entry to the survey required endorsement of four consent statements (“I have read and understood the above information”, “I voluntarily agree to participate”, “I understand that I may withdraw at any time”, and “I agree that my anonymous data may be used for academic research”).

The survey was anonymous and did not collect identifying information. Data were used solely for research purposes and reported in aggregate. The questionnaire contained five sections: (1) demographics and screening items for fitness app use (non-users were automatically terminated); (2) fitness app use scale (7 items); (3) International Physical Activity Questionnaire–Short Form (IPAQ-SF) assessing physical activity in the past 7 days; (4) flow experience scale (36 items) referring to the most recent app-supported exercise session; and (5) exercise motivation scale (24 items). All scales were administered in Likert-type formats. Quality control procedures included: (1) an embedded attention-check item; (2) removal of cases with completion times <3 min or >60 min; (3) restriction by IP address to prevent duplicate submissions; and (4) exclusion of responses showing clear patterns. Data transmission was encrypted; storage was password-protected, with access restricted to the research team.

To improve the language and readability of this manuscript, ChatGPT-4 (OpenAI) was used for English language editing assistance. All AI-assisted content was subsequently reviewed, revised, and verified by the authors, who take full responsibility for the accuracy and integrity of the final manuscript.

### 2.3 Measures

#### 2.3.1 Fitness app use

Fitness app use was assessed using a self-developed scale informed by prior literature on mobile health behavior and tailored to university students’ app-use characteristics. The scale is conceptualized as a *unidimensional* composite reflecting overall app use intensity, encompassing five content domains across 7 items: (1) *usage frequency* (e.g., “I use a fitness app almost every day”); (2) *usage duration* (e.g., “My usage time per session is usually long”); (3) *feature engagement* (e.g., “I frequently check my exercise data in the app”; “I use workout plans or guided courses within the app”); (4) *perceived dependence* (e.g., “Fitness apps have become an indispensable part of my exercise life”; “I rely on the app to set and accomplish my exercise goals”); and (5) *immersive engagement* (e.g., “Using the app makes me more engaged and focused during exercise”). Items were rated on a 5-point Likert scale (1 = strongly disagree to 5 = strongly agree). Because the construct is operationalized as a single global dimension, total scores were computed by summing all item responses, with higher scores indicating greater overall app use intensity. Given the unidimensional structure, exploratory or confirmatory factor analysis was not required; the coherence of the composite is instead supported by strong internal consistency (Cronbach’s α=0.871), which is consistent with a single-factor scale of this length.

#### 2.3.2 Flow experience

Flow experience was measured using the Chinese revised version of the Flow State Scale-2 (FSS-2) [27]. The instrument includes 36 items assessing flow during participants’ most recent fitness app–supported exercise session across nine dimensions: challenge–skill balance, action–awareness merging, clear goals, unambiguous feedback, concentration on the task at hand, sense of control, loss of self-consciousness, transformation of time, and autotelic experience (4 items per dimension) [28, 29]. Items were rated on a 5-point Likert scale (1 = strongly disagree to 5 = strongly agree). Item scores were summed, with higher totals indicating stronger flow. In the present study, internal consistency was excellent (Cronbach’s α=0.985).

#### 2.3.3 Exercise motivation

Exercise motivation was assessed using the Chinese revised version of the Behavioral Regulation in Exercise Questionnaire-2 (BREQ-2) [30], which has been widely applied in Chinese samples [31]. The scale contains 24 items covering five regulatory styles: amotivation, external regulation, introjected regulation, identified regulation, and intrinsic motivation (4 items each). Items were rated on a 5-point scale (0 = not true for me to 4 = very true for me). Following self-determination theory, autonomous motivation was operationalized using the Relative Autonomy Index (RAI): RAI = (2× intrinsic motivation) + identified regulation − introjected regulation − (2× external regulation). Higher RAI scores indicate stronger autonomous motivation. RAI scores can theoretically range from −18 to +14, with positive values reflecting stronger autonomous motivation and negative values indicating more controlled motivational regulation. Internal consistency in the present study was excellent (Cronbach’s α=0.940).

#### 2.3.4 Physical activity behavior

Given the absence of a validated scale specifically designed to measure exercise behavior as defined in this study, physical activity assessed via the IPAQ-SF was used as the primary behavioral outcome and operational proxy for exercise behavior. This approach is consistent with established practice in the exercise psychology literature, where objectively scorable physical activity measures are widely employed as behavioral indicators of exercise engagement. Physical activity was measured using the Chinese version of the International Physical Activity Questionnaire–Short Form (IPAQ-SF) [32]. The IPAQ-SF assesses vigorous activity, moderate activity, walking, and sedentary time over the past 7 days. Participants reported the number of days and average minutes per day for each activity category. Following standard scoring procedures, activity minutes were converted to metabolic equivalent task minutes (MET-min) using MET values of 8.0 (vigorous), 4.0 (moderate), and 3.3 (walking). Total physical activity was computed as: Total MET-min = (vigorous days × vigorous minutes × 8.0) + (moderate days × moderate minutes × 4.0) + (walking days × walking minutes × 3.3). Higher total MET-min indicates greater physical activity.

### 2.4 Data analysis

All analyses were conducted using Stata 17.0. Missing data were handled via complete-case analysis. Outliers were identified and removed using boxplots and standardized residuals (∣Z∣>3). Normality was evaluated using skewness and kurtosis criteria (∣skewness∣<2, ∣kurtosis∣<7) and the Shapiro–Wilk test. Common method bias was assessed using Harman’s single-factor test; bias was considered limited when the first factor accounted for <40% of the total variance. Multicollinearity was evaluated using variance inflation factors (VIF < 5) and tolerance (Tolerance > 0.20).

Primary analyses included: (1) descriptive statistics (means, standard deviations, skewness, kurtosis); (2) Pearson correlation analyses; (3) independent-samples t tests and one-way analyses of variance to examine group differences across demographic variables; (4) hierarchical regression analyses to test direct and mediation effects; and (5) bootstrapped mediation analyses using 5,000 resamples with bias-corrected percentile 95% confidence intervals, with mediation inferred when the confidence interval excluded zero. Tested pathways included: fitness app use → flow experience → physical activity, fitness app use → exercise motivation → physical activity, and the sequential (chain) pathway fitness app use → flow experience → exercise motivation → physical activity. Structural equation modeling was used to evaluate the theoretical model, with model fit assessed using $\chi^{2}/df<3$, RMSEA < 0.08, CFI > 0.90, TLI > 0.90, and SRMR < 0.08. Statistical significance was set at α=0.05 (two-sided) [33].

## 3 Results

### 3.1 Assessment of common method bias and data quality

Prior to hypothesis testing, we conducted a systematic evaluation of data quality. Common method bias was examined using Harman’s single-factor test. All scale items (67 items in total) were entered into an exploratory factor analysis, and the first unrotated factor was extracted using principal component analysis. The first factor had an eigenvalue of 27.99 and accounted for 41.78% of the total variance, marginally exceeding the conventional 40% criterion [34]. Although this slightly elevated proportion warrants acknowledgment, it is widely recognized that Harman’s single-factor test is a conservative and imperfect diagnostic rather than a definitive indicator of bias [34]. Multiple procedural safeguards were implemented to mitigate common method variance: the survey was administered anonymously, scale items were randomized in presentation order, reverse-scored items were included, and participants completed separate validated instruments rather than a single omnibus questionnaire. Collectively, these measures substantially reduce the risk of common method bias inflating the observed associations, and we consider its influence on the substantive conclusions to be limited. Nonetheless, the marginally elevated Harman value (41.78%) should be acknowledged as a study limitation; future work is encouraged to supplement Harman’s test with a common latent factor (CLF) approach within structural equation modeling—in which an orthogonal CLF is constrained to load on all indicators—to provide a more rigorous statistical estimate of shared method variance and to verify that substantive path coefficients remain stable after partialling out method effects [34].

Normality diagnostics indicated that the focal study variables exhibited acceptable distributional properties, with skewness ranging from −0.41 to 0.58 (absolute values < 2) and kurtosis ranging from −0.73 to 0.92 (absolute values < 7), supporting the normality assumption. Multicollinearity was assessed using variance inflation factors (VIF) and tolerance statistics. All predictors showed VIF values below 3 (flow experience: 2.39; fitness app use: 1.96; autonomous motivation: 1.38; mean VIF: 1.91), and tolerance values exceeded 0.40 (range: 0.419–0.725), indicating no serious multicollinearity concerns [35]. Collectively, these checks suggest that the dataset is of adequate quality and suitable for subsequent statistical analyses.

### 3.2 Descriptive statistics and correlation analyses

Table 1 summarizes the descriptive statistics and Pearson correlation coefficients for the study variables. The sample comprised 350 university students. Fitness app use (M=3.73, SD=0.67), flow experience (M=3.01, SD=0.73), and autonomous motivation (M=3.86, SD=8.26) were all at a moderate-to-relatively-high level. Mean weekly physical activity expenditure was 2,891.90 MET-min/week (SD=1,979.09).

**Table 1:** Descriptive statistics and correlations among study variables.

Variable	M	SD	1	2	3	4
1. Fitness app use	3.73	0.67	–			
2. Flow experience	3.01	0.73	.650***	–		
3. Autonomous motivation	3.86	8.26	.043	.425***	–	
4. Exercise behavior (METs)	2,891.90	1,979.09	.554***	.667***	.317***	–

N=350. ***p<.001. Pearson correlations are presented.

Correlation analyses indicated that fitness app use was positively associated with flow experience (r=0.650, p<0.001) and physical activity (r=0.554, p<0.001), whereas its association with autonomous motivation was not statistically significant (r=0.043, ns). Flow experience was positively correlated with autonomous motivation (r=0.425, p<0.001) and physical activity (r=0.667, p<0.001). Overall, the directions of the observed associations were consistent with the hypothesized relationships, providing preliminary support for subsequent model testing.

### 3.3 Tests of the moderated mediation model

To test the hypotheses, we conducted moderated mediation analyses using Hayes’ PROCESS framework [20]. All continuous variables were standardized prior to analysis to facilitate interpretation and to reduce potential multicollinearity. Hierarchical regression results are reported in Table 2.

**Table 2:** Hierarchical regression analysis for moderated mediation model.

	Model 1	Model 2	Model 3
Predictor	Flow experience	Exercise behavior	Exercise behavior
Step 1: main effects			
Fitness app use (X)	0.598*** (0.026)	0.208*** (0.045)	0.554*** (0.044)
Autonomous motivation (W)	0.339*** (0.029)	–	–
Flow experience (M)	–	0.531*** (0.052)	–
Step 2: interaction			
X×W	0.423*** (0.027)	–	–
Model fit			
$R^{2}$	0.743	0.469	0.307
Adjusted $R^{2}$	0.740	0.466	0.305
F	420.81***	128.89***	160.64***

N=350. Standardized regression coefficients are presented, with robust standard errors in parentheses. Model 1 tests the moderation effect (outcome: flow experience). Model 2 tests the mediation model (outcome: physical activity; mediator included). Model 3 estimates the total effect (outcome: physical activity; mediator excluded). *** p<.001.

#### 3.3.1 Mediation effect test (H1)

Model 2 assessed whether flow experience mediated the association between fitness app use and physical activity. Flow experience was positively associated with physical activity (β=0.531, SE=0.052, t=10.18, p<0.001). The direct effect of app use on physical activity remained significant after accounting for flow (β=0.208, SE=0.045, t=4.58, p<0.001). This model explained 46.9% of the variance in physical activity ($R^{2}=0.469$, F(2,347)=128.89, p<0.001).

Bootstrapping with 5,000 resamples was used to test the indirect effect. As shown in Table 3, the indirect effect of app use on physical activity through flow was significant [indirect effect = 0.318, SE=0.034, 95% CI (0.254, 0.384)]. The indirect effect accounted for 60.4% of the total effect, whereas the direct effect accounted for 39.6%. Because both direct and indirect effects were significant, the findings supported a partial mediation model, consistent with H1.

**Table 3:** Effect decomposition and mediation analysis.

Effect path	β	SE	95% CI	% of Total
Direct effect ($c^{\prime }$): X→Y	0.208***	0.045	[0.119, 0.297]	39.6%
Indirect effect (a×b): X→M→Y	0.318***	0.034	[0.254, 0.384]	60.4%
Total effect (c): X→Y	0.526***	0.044	[0.468, 0.640]	100.0%
Mediation type	Partial mediation			

N=350. Indirect effects were estimated with 5,000 bootstrap resamples. X= fitness app use; M= flow experience; Y= exercise behavior. *** p<.001.

#### 3.3.2 Moderation test (H2)

**Figure 1:**
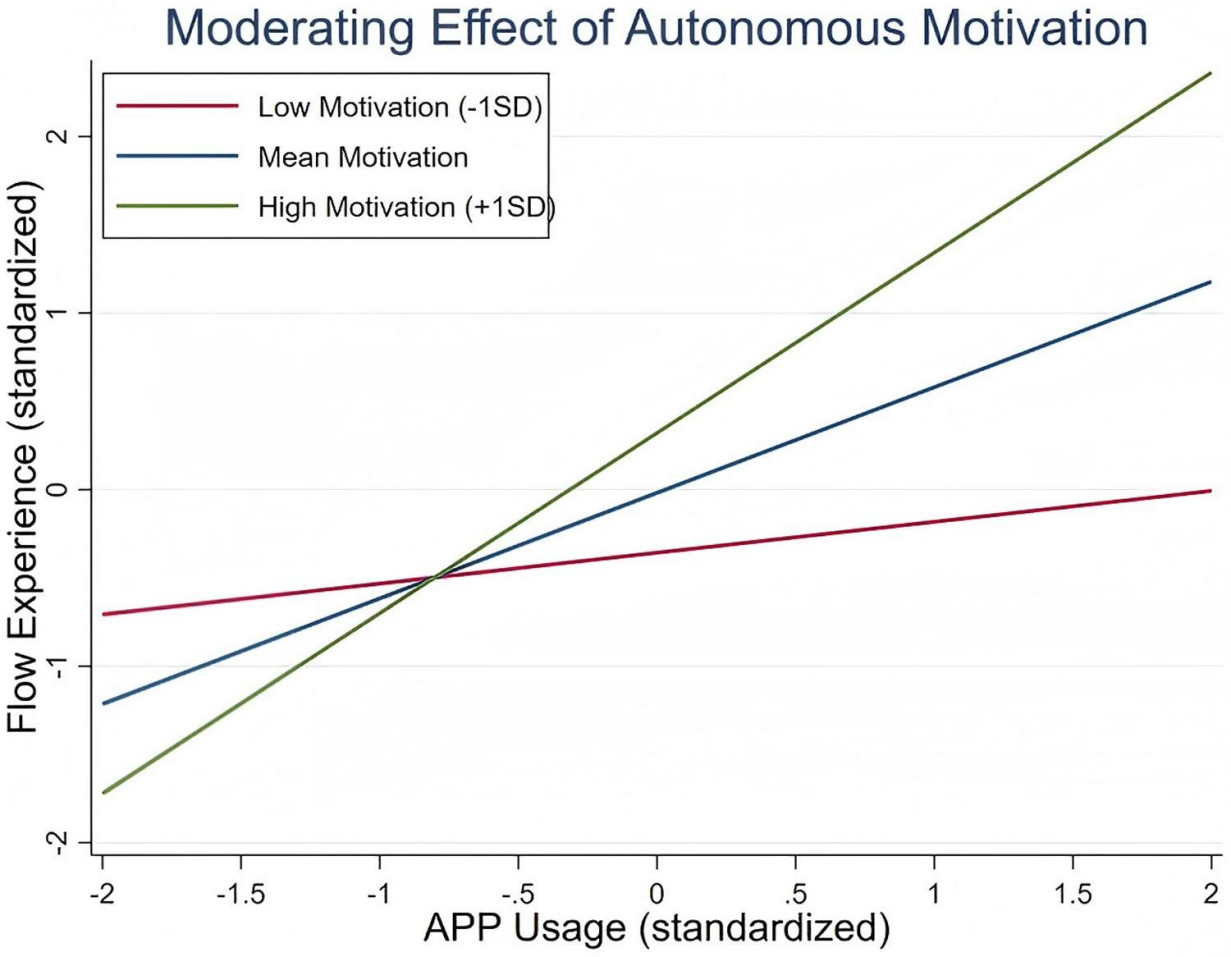
Moderating effect of autonomous motivation on the relationship between app use and flow experience. Note: The three lines in the figure represent three levels of autonomous motivation: low (1 SD below the mean), medium (mean), and high (1 SD above the mean). All slopes differ significantly from zero (p<0.001). The interaction effect is significant (β=0.423, p<0.001), indicating that autonomous motivation strengthens the positive relationship between fitness app use and flow experience.

Model 1 examined whether autonomous motivation moderated the association between fitness app use and flow experience. The interaction term (X×W) was statistically significant (β=0.423, SE=0.027, t=15.56, p<0.001), supporting H2. The model explained 74.3% of the variance in flow experience ($R^{2}=0.743$, F(3,346)=420.81, p<0.001). Adding the interaction increased explained variance by 18.1% ($\Delta R^{2}=0.181$, p<0.001), indicating a substantively meaningful moderation effect. Multicollinearity diagnostics suggested no concerns (mean VIF = 1.02; range: 1.01–1.03). To interpret the interaction, simple slope analyses were conducted at three levels of autonomous motivation (low: −1 SD; mean: 0; high: +1 SD). As shown in Table 4, Panel A and Figure 1, the positive association between app use and flow experience was stronger at high autonomous motivation (β=1.021, SE=0.038, p<0.001) and weaker at low autonomous motivation (β=0.175, SE=0.037, p<0.001). The difference between the slopes was large and statistically significant (Δβ=0.846, SE=0.054, p<0.001), indicating that students with higher autonomous motivation tended to experience markedly greater flow when engaging with fitness apps.

**Table 4:** Conditional effects and moderated mediation analysis.

Panel A: simple slopes analysis (X→M)					
Level of autonomous motivation	β	SE	t	95% CI	
Low (−1 SD)	0.175***	0.037	4.73	[0.102, 0.248]	
Mean (0)	0.598***	0.026	22.84	[0.547, 0.649]	
High (+1 SD)	1.021***	0.038	26.87	[0.946, 1.096]	
Difference (High − Low)	0.846***	0.054	15.56	[0.739, 0.953]	
Panel B: conditional indirect effects (X→M→Y)					
Level of autonomous motivation	Indirect effect	Boot SE	Boot 95% CI		% of Total
Low (−1 SD)	0.093	0.026	[0.044, 0.147]		17.7%
Mean (0)	0.318	0.034	[0.254, 0.384]		60.4%
High (+1 SD)	0.543	0.043	[0.462, 0.630]		103.2%${}^{\text{a}}$
Index of moderated mediation	0.225	0.033	[0.162, 0.292]		–

N=350. Panel A presents conditional effects of app use on flow experience (the X→M path). Panel B reports conditional indirect effects via flow based on 5,000 bootstrap resamples. All confidence intervals exclude zero, indicating statistically significant effects.

${}^{\text{a}}$ In the high-autonomous-motivation condition, the indirect effect slightly exceeded the total effect, consistent with a suppression (inconsistent mediation) pattern. *** p<.001.

#### 3.3.3 Moderated mediation test (H3)

Conditional indirect effects at different levels of autonomous motivation are reported in Table 4, Panel B. The indirect effect increased as autonomous motivation increased. At low autonomous motivation, the indirect effect was weakest [β=0.093, 95% CI (0.044, 0.147)]; at the mean level, it was moderate [β=0.318, 95% CI (0.254, 0.384)]; at high autonomous motivation, it was strongest [β=0.543, 95% CI (0.462, 0.630)].

The index of moderated mediation was significant [Index = 0.225, SE=0.033, 95% CI (0.162, 0.292)], providing formal evidence that autonomous motivation moderated the strength of the indirect pathway. In practical terms, the indirect effect in the high-autonomous-motivation group was 5.84 times that in the low-autonomous-motivation group (0.543/0.093), indicating that the mechanism linking app use to physical activity via flow was substantially amplified among students higher in autonomous motivation. H3 was supported.

### 3.4 Robustness checks

Several additional analyses were conducted to evaluate robustness. Standardized residual diagnostics suggested no extreme outliers (all ∣z∣<3.29). Cook’s distances were below the conventional cutoff (4/n=0.011), indicating no highly influential cases. Model assumptions were generally well satisfied; residuals were approximately normally distributed and did not indicate problematic heteroscedasticity. Sensitivity analyses controlling for demographic variables (gender and year of study) produced results that were substantively consistent with the primary analyses, supporting the stability of the findings.

## 4 Discussion

This study examined how fitness app use is linked to university students’ physical activity through flow experience, and whether autonomous motivation conditions this process. Using a cross-sectional survey of 350 university students who reported current use of fitness apps, we tested the proposed relationships via structural equation modeling and moderated mediation analysis. The results indicate that fitness app use was positively associated with physical activity through the mediating role of flow experience, with the indirect pathway accounting for 60.4% of the total effect. Autonomous motivation significantly moderated the association between app use and flow experience (β=0.423, p<0.001) and, consequently, moderated the strength of the indirect pathway from app use to physical activity via flow.

### 4.1 The direct association between fitness app use and physical activity

A robust positive association emerged between fitness app use and physical activity (r=0.554, p<0.001). Importantly, this relationship remained significant after accounting for flow experience (β=0.208, p<0.001), suggesting that fitness apps are linked to physical activity not only through experiential mechanisms, but also through more direct behavioral supports. This pattern is broadly consistent with evidence syntheses showing that app-based and tracker-supported interventions can yield positive, albeit heterogeneous, effects on health behavior and physical activity outcomes [36, 37].

From a behavioral perspective, fitness apps can reduce both practical and psychological barriers to exercise by supporting structured self-monitoring, translating vague intentions into quantifiable goals, and providing immediate feedback. Social features may further supply encouragement, accountability, and normative cues, which are especially relevant in student populations where peer influence is salient. Evidence integrating technology acceptance perspectives indicates that when users perceive an app as useful and easy to use, continued engagement becomes more likely, thereby increasing the probability of downstream behavioral benefits [38]. In concrete terms, streamlined interfaces, low-friction logging, intuitive data visualization (e.g., energy expenditure trends and route maps), and personalization functions can strengthen perceived usefulness and usability, which in turn may stabilize use and facilitate the maintenance of activity routines.

The behavioral change wheel framework provides a complementary explanation by emphasizing that effective interventions typically operate through capability, opportunity, and motivation [39]. Fitness apps can contribute to capability via instructional videos and technique guidance, expand opportunity via short-session workouts and home-based programs, and influence motivation through gamified elements and social comparison. Gamification appears particularly relevant for engagement and persistence: systematic reviews in eHealth suggest that points, badges, and leaderboards can increase participation and adherence [40], and longitudinal evidence during the COVID-19 period indicates that more frequent app use was associated with more favorable changes in physical activity, with gamified features playing a buffering role against activity declines [41]. At the same time, an overreliance on external rewards may carry longer-term risks if engagement is sustained primarily by incentives that may lose salience over time. In the present data, the attenuation from the total effect to the direct effect, once flow was included, indicates that a substantial portion of the app–activity association operates through psychological pathways—consistent with the view that digital tools are most likely to support durable behavior change when they systematically reshape users’ experience and motivational dynamics, rather than merely adding prompts or rewards.

### 4.2 The mediating role of flow experience

The present findings indicate that flow experience serves as a significant mediator linking fitness app use to physical activity [indirect effect = 0.318, 95% CI (0.254, 0.384)], accounting for 60.4% of the total effect. This pattern underscores a point that is easy to overlook in technology-enabled intervention work: providing feature-rich digital tools is not enough; what matters is whether the tool reliably produces a usage context capable of eliciting an optimal psychological experience. Flow—characterized by deep absorption, sustained attentional focus, and enjoyment during the activity—has long been regarded as a key experiential driver of continued participation and intrinsic motivation. Event-focused interview evidence portrays flow as an enjoyable, intrinsically rewarding state marked by intense engagement with the focal activity, reduced intrusion of irrelevant thoughts and affect, and an experiential sense that the activity “fits” or unfolds smoothly [42]. The nine-dimension model of flow further clarifies that flow is most likely when skills are well matched to task challenges, goals are clear, and feedback is immediate and unambiguous [14].

Consistent with this framework, students reporting greater fitness app use were more likely to experience flow during app-supported exercise (r=0.650, p<0.001), and flow in turn was positively associated with physical activity (β=0.531, p<0.001). This mediational mechanism converges with evidence from wearable-technology research. For example, among smartwatch users, device characteristics such as interactivity, autonomy support, convenience, and novelty have been shown to promote exercise continuance intentions indirectly by enhancing flow—particularly the absorption component, which exhibits a statistically meaningful association with intention [43]. Fitness apps can instantiate the core antecedents of flow in concrete and repeatable ways. Personalised training plans can adjust intensity and difficulty in line with users’ current fitness, maintaining a dynamic challenge–skill balance; running apps, for instance, can recalibrate targets in real time based on pace and heart-rate data, reducing the likelihood of boredom from under-challenge or anxiety from over-challenge. Meanwhile, immediate feedback—delivered through voice prompts, haptic cues, and real-time dashboards—keeps users continuously informed about performance, directly aligning with the requirement for clear and timely feedback.

Flow also carries a distinctive reinforcement profile. When individuals experience flow while exercising, they typically report heightened pleasure, a sense of control, and autotelic satisfaction, such that the activity becomes rewarding in its own right rather than merely instrumental to distal health outcomes. A systematic review synthesising affect–activity links reported that positive affect during moderate-intensity exercise is meaningfully associated with subsequent physical activity, whereas post-exercise affect shows near-zero associations—suggesting that in-the-moment experience may be more consequential for future participation than how one feels afterward [44]. Recent work on engagement with health and fitness apps likewise suggests that immersive experiences can energize motivation by directing attention toward self-improvement and perceived achievement within a digitally mediated environment, thereby strengthening intentions to continue use through enhanced intrinsic motivation [45]. Evidence from large-sample research on Chinese fitness app users further indicates that sustained app use is associated with improvements in physical, emotional, social, and cognitive states, with potential benefits for overall well-being [21].

A further detail in the present data is telling: the correlation between flow experience and physical activity (r=0.667) exceeded that between app use and physical activity (r=0.554). While correlational comparisons should not be over-interpreted, the pattern is nonetheless consistent with the claim that flow occupies a more proximal position in the pathway to sustained activity. Practically, this implies that app developers and exercise intervention designers should move beyond a checklist view of features and treat flow facilitation as a primary design objective. Concretely, this entails careful calibration of challenge to skill, explicit and personally meaningful goal structures, immediate and interpretable feedback, and interface and interaction designs that support immersion rather than fragment attention.

### 4.3 The moderating role of autonomous motivation

The most novel contribution of this study lies in the clear moderating effect of autonomous motivation on the association between fitness app use and flow experience (β=0.423, p<0.001), as well as the resulting moderated mediation [index of moderated mediation = 0.225, 95% CI (0.162, 0.292)]. The implication is not subtle: users do not benefit from fitness apps to the same degree, and motivational orientation functions as a boundary condition that shapes whether—and how—technology translates into an optimal exercise experience. Simple slope analyses make this heterogeneity concrete. Among students with high autonomous motivation (+1 SD), app use strongly facilitated flow (β=1.021, p<0.001); among those with low autonomous motivation (−1 SD), the same association was markedly weaker (β=0.175, p<0.001). The difference in effect magnitude reached 5.84-fold, suggesting that autonomous motivation is not merely a modest interaction term but a structurally important determinant of the intervention pathway.

This moderation can be interpreted through core propositions of self-determination theory and adjacent models of motivational user experience. The METUX framework emphasizes that digital technologies influence well-being to the extent that they support (rather than thwart) the basic psychological needs for autonomy, competence, and relatedness [46]. Individuals high in autonomous motivation tend to construe exercise as a vehicle for self-expression and value realization, which in turn changes the meaning of app functions: goal-setting is more likely to be experienced as self-endorsed direction rather than imposed demands, and performance feedback is more readily interpreted as information for self-improvement rather than as external evaluation. Once that interpretive frame is in place, deeper engagement becomes more probable, and the app’s affordances (clear goals, immediate feedback, adjustable challenge) can more readily crystallize into flow.

By contrast, individuals low in autonomous motivation—often driven by external pressures or guilt (e.g., social expectations, health anxiety)—may perceive the same app features as monitoring and judgment. Under that appraisal, notifications can feel like coercive prompts, rankings may induce anxiety rather than energize effort, and gamified elements can register as superficial rather than enjoyable. Qualitative evidence among fitness app users indicates that perceived autonomy satisfaction is pivotal for continued app engagement, whereas perceived controlling features reliably undermine motivation and reduce willingness to persist [47]. From the perspective of the moderated mediation model, the current data align tightly with this account: the conditional indirect effect via flow was strongest at high autonomous motivation [β=0.543, 95% CI (0.462, 0.630)] and weakest at low autonomous motivation [β=0.093, 95% CI (0.044, 0.147)], again reflecting a 5.84-fold difference in magnitude. This is a practically consequential result, because it implies that the effectiveness of app-based approaches depends heavily on the motivational composition of the target population. Evidence from emerging adults similarly highlights that motivational variables and related self-regulatory beliefs meaningfully predict physical activity and body composition, reinforcing the centrality of motivation type in exercise behavior [48]. In applied terms, fitness apps may efficiently amplify flow and behavior among individuals already high in autonomous motivation, whereas for those lacking such motivation, the marginal benefit of “more technology” is likely limited; achieving comparable gains may require pairing app use with motivation-building interventions that explicitly support autonomy and competence rather than relying on monitoring, comparison, or external incentives.

### 4.4 Moderated mediation: an integrative account

By integrating fitness app use, flow experience, autonomous motivation, and physical activity within a moderated mediation framework, the present study clarifies when and through what pathway app use is most likely to translate into behavioral change. The conditional indirect effect via flow differed substantially across levels of autonomous motivation: at high autonomous motivation (+1 SD), the indirect effect was 0.543 [95% CI (0.462, 0.630)], whereas at low autonomous motivation (−1 SD) it dropped to 0.093 [95% CI (0.044, 0.147)]. The index of moderated mediation was statistically significant [0.225, 95% CI (0.162, 0.292)], indicating that autonomous motivation reliably conditions the mediational pathway through flow. This pattern is consistent with the logic of conditional indirect effects, in which the magnitude of mediation is contingent on a moderator rather than assumed to be constant across persons [49].

Theoretically, these findings sharpen how digital health interventions should be understood: design features such as goal setting, planning, self-monitoring, prompts/cues, and personalization may be generally “useful,” but their capacity to generate the experiential engine of behavior change—here, flow—appears motivationally gated [50]. Put differently, the same technical affordances can be metabolized differently depending on whether users approach exercise from a self-endorsed (autonomous) stance or from pressure- and guilt-laden regulation. This is the point at which a person–tool fit perspective becomes more than a slogan. The results imply that intervention efficacy is partly a matching problem: the app is not simply a uniform stimulus; it is an environment whose psychological meaning depends on the user’s motivational orientation.

This conclusion aligns with the broader turn toward precision health in physical-activity promotion, which argues for tailoring prescriptions to multidimensional baseline phenotypes, including psychological factors [51]. Evidence syntheses on continued use of mobile fitness apps likewise highlight satisfaction, perceived usefulness, perceived enjoyment, and social influence as central determinants, while also noting that mechanisms vary across user subgroups [52]. In the present data, one can see how “similar mechanisms” can still yield sharply different outcomes: although the proportionate transmission from app use to behavior via flow looks comparable across motivational strata (e.g., 0.543/1.021 ≈ 0.532 vs. 0.093/0.175 ≈ 0.531), the markedly different strength of the X→M link produces large absolute differences in downstream behavioral impact. Practically, this favors a staged and segmented strategy: for users already high in autonomous motivation, app-based personalization and feedback may efficiently amplify flow and activity; for users low in autonomous motivation, introducing the same tool without prior motivational work is likely to have low yield, making autonomy-supportive motivational enhancement (e.g., motivational interviewing, values clarification, reframing metrics as informational rather than evaluative) a plausible prerequisite rather than an optional add-on.

## 5 Limitations and future directions

Several limitations warrant consideration. The cross-sectional design constrains causal inference: although fitness app use, flow experience, autonomous motivation, and physical activity were significantly associated, the temporal ordering of these relationships cannot be established. Future work should employ longitudinal and intensive repeated-measures designs to examine dynamic coupling over time, with particular attention to whether flow has cumulative effects and whether motivational internalization unfolds progressively rather than discretely. A second limitation is the reliance on self-report measures, which may introduce social desirability and recall bias; subsequent research could strengthen ecological validity by triangulating data sources, for example by extracting backend app logs (frequency, duration, feature use), using wearable devices to capture objectively measured activity, and adopting experience sampling methods (ESM) to assess momentary flow during exercise episodes. Third, the sample comprised only university students, which limits generalizability to other age groups, occupations, fitness levels, and cultural contexts; replication in more heterogeneous populations is therefore necessary to evaluate boundary conditions of the proposed mechanism. Fourth, the present model focused on flow and autonomous motivation; future studies could test alternative mediators and moderators—such as social support, self-efficacy, perceived autonomy support from the app, and technology acceptance variables—to map competing or complementary pathways. Comparing flow experience against these alternative mechanisms in a single model would clarify the unique vs. shared explanatory contributions of each mediator: for instance, perceived enjoyment and perceived usefulness may capture overlapping but distinct aspects of the app–activity association that flow alone cannot fully account for, and self-efficacy may operate in parallel as a motivational resource shaping persistence beyond any single exercise session [19]. Fifth, the fitness app use scale was purpose-built for this study and has not yet undergone independent psychometric validation; while strong internal consistency (α=0.871) supports the reliability of the unidimensional composite, future research should conduct formal confirmatory factor analysis to establish construct validity before the scale is adopted in other samples or contexts. Sixth, the present study did not examine potential heterogeneity across demographic subgroups. Given that gender, year of study, app usage duration, and habitual exercise frequency may meaningfully shape how individuals engage with fitness apps and experience flow, future studies are encouraged to conduct multi-group moderated mediation analyses comparing these subgroups—for example, male vs. female students and short-term vs. long-term app users—to identify boundary conditions of the proposed mechanism and to inform more targeted app design recommendations. Finally, while the moderated mediation mechanism was statistically supported, the study did not directly test intervention efficacy. Future research should translate these findings into experimental designs, such as developing motivation-stratified personalization features or autonomy-supportive modules for low-autonomous-motivation users, and evaluating their long-term effectiveness via randomized controlled trials.

## 6 Conclusion

This study provides a systematic account of how fitness app use relates to university students’ physical activity by integrating flow theory and self-determination theory within a moderated mediation framework. The findings indicate that fitness app use not only exhibited a direct positive association with physical activity, but was also indirectly associated with activity through flow experience, which functions as a central experiential mechanism. More critically, autonomous motivation emerges as a decisive boundary condition: among students higher in autonomous motivation, app use is more likely to translate into stronger flow and, consequently, greater physical activity, whereas the corresponding pathway is substantially weaker for those lower in autonomous motivation. This motivational contingency implies that a single technological solution is unlikely to be universally effective across users. Theoretically, the study advances digital health behavior-change research by specifying an experience-based mechanism whose strength depends on motivational orientation; practically, it argues for “precision” in app design and dissemination, in which user segmentation and autonomy-supportive motivational enhancement may be necessary—especially for low-autonomous-motivation users—before technical affordances can reliably yield sustained behavioral change.

## Data Availability

The datasets generated and/or analyzed during the current study are available in the Figshare repository: https://doi.org/10.6084/m9.figshare.31292512.
